# Promoting Healthy Diet, Physical Activity, and Life-Skills in High School Athletes: Results from the WAVE Ripples for Change Childhood Obesity Prevention Two-Year Intervention

**DOI:** 10.3390/nu10070947

**Published:** 2018-07-23

**Authors:** Yu Meng, Melinda M. Manore, John M. Schuna, Megan M. Patton-Lopez, Adam Branscum, Siew Sun Wong

**Affiliations:** 1 School of Biological and Population Health Sciences, College of Public Health and Human Sciences, Oregon State University, Corvallis, OR 97330, USA; melinda.manore@oregonstate.edu (M.M.M.); John.Schuna@oregonstate.edu (J.M.S.J.); Adam.Branscum@oregonstate.edu (A.B.); 2 Division of Health and Exercise Science, Western Oregon University, 240 Richard Woodcock Education Center, 345 Monmouth Ave N., Monmouth, OR 97361, USA; pattonlm@wou.edu; 3 Family and Community Health, School of Biological and Population Health Sciences, College of Public Health and Human Sciences, Oregon State University, Corvallis, OR 97330, USA; siewsun.wong@oregonstate.edu

**Keywords:** adolescent, free or reduced lunch, National School Lunch Program, Fitbit, soccer, low-income, Latino, added sugar, sport nutrition, sport

## Abstract

The purpose of this study was to compare changes in diet and daily physical activity (PA) in high school (HS) soccer players who participated in either a two-year obesity prevention intervention or comparison group, while controlling for sex, race/ethnicity, and socioeconomic status. Participants (*n* = 388; females = 58%; Latino = 38%; 15.3 ± 1.1 years, 38% National School Breakfast/Lunch Program) were assigned to either an intervention (*n* = 278; 9 schools) or comparison group (*n* = 110; 4 schools) based on geographical location. Pre/post intervention assessment of diet was done using Block Fat/Sugar/Fruit/Vegetable Screener, and daily steps was done using the Fitbit-Zip. Groups were compared over-time for mean changes (post-pre) in fruit/vegetables (FV), saturated fat (SF), added sugar, and PA (daily steps, moderate-to-vigorous PA) using analysis of covariance. The two-year intervention decreased mean added sugar intake (−12.1 g/day, CI (7.4, 16.8), *p* = 0.02); there were no differences in groups for FV or SF intake (*p* = 0.89). For both groups, PA was significantly higher in-soccer (9937 steps/day) vs. out-of-soccer season (8117 steps/day), emphasizing the contribution of organized sports to youth daily PA. At baseline, Latino youth had significantly higher added sugar intake (+14 g/day, *p* < 0.01) than non-Latinos. Targeting active youth in a diet/PA intervention improves diet, but out of soccer season youth need engagement to maintain PA (200).

## 1. Introduction

Currently, 20.6% of United States (US) adolescents are obese [1]. Reducing obesity is best accomplished through prevention efforts, yet the practices and methods to implement this goal are still emerging. In 2017, 52.7% of public high school (HS) students participated in sports [2,3]. Since many youth begin playing sports at a young age and continue through HS, this time may represent a window of opportunity to begin building life-skills that emphasize daily physical activity (PA), and eating a healthy diet to fuel growth, performance and health. Thus, targeting youth involved in HS school sports for obesity prevention, may be an overlooked opportunity to engage these youth. Diet and PA are two key lifestyle factors in preventing and reducing obesity risk. Developing these life-skills as part of the youth sport experience will benefit participants for a lifetime, long after school sports are over. 

For US youth, soccer is one of the fastest growing sports and among one of the top five most popular games for male and female youth. Since 2000, over three million youth have played soccer annually [4]. Soccer is especially popular among Latino youth [5]. Although Latina girls have lower sport participation rates than their peers, soccer is the one sport they are most likely to play [5]. Based on data from the National Federation of State High School Associations, more than eight million adolescents participated in HS sports in 2016–2017 [3], yet there is limited nutrition education given to these youth related to diet and PA. We recently examined the sport nutrition knowledge, attitudes and beliefs of HS soccer players. Results show that sports nutrition knowledge is low (46%), especially in Latino youth (39%) and those participating in National School Breakfast/Lunch Program (NSLP) (41%) [6].

Children and adolescents with lower social economic status have higher obesity rates, poorer diets, and lower levels of PA [7,8,9]. Latino youth are especially at risk for obesity [10], with significantly higher obesity prevalence (25.8%) than non-Latino youth (11.0–22.0%) [1]. For Latino youth, obesity rates are high regardless of social economic status [8]. Khan et al. found higher body mass index (BMI, kg/m^2^) levels among second-generation Mexican-American adults compared with those born in Mexico [11]. This increase in body weight may be due to the availability of cheap food that can be higher in added sugar and fat than healthier foods (e.g., fruit/vegetable (FV), whole grains, low-fat dairy/meats) [12]. Finally, Boutelle et al. [13] found that Latino parents with HS adolescents were more likely than White, Asian, and Black families to purchase fast-food for the family meals (>3 time/week). No study has examined the influences of parental practices on HS soccer players’ diet and PA.

Promoting a healthy diet, daily PA, and life-skills through sport programs provides an opportunity to reach groups of diverse adolescents. Youth athletes see these programs as ‘improving sport performance’ and less as ‘another classroom lesson’. The WAVE~Ripples for Change program was developed for HS soccer players to encourage PA outside of sport, and to teach nutrition and life-skills (e.g., meal planning, shopping on a budget, food preparation/cooking skills, and gardening) to support sustainable healthy eating and lifetime PA. Thus, the purpose of this study was to compare changes in diet and daily PA in HS soccer players who participated in either a two-year obesity prevention intervention or comparison group, while controlling for sex, race/ethnicity, and socioeconomic status. In order to examine the influences of parental practices on HS soccer player’s diet and PA, youth’s self-report of their parents’ involvement in healthy eating (e.g., modeling, setting rules and providing healthy food at home), setting screen time limits, and modeling PA were compared to youth’s self-reported FV, saturated fat, added sugar intakes, and measured PA using Fitbit-zip.

## 2. Materials and Methods

### 2.1. Program Overview and Experimental Design

**Figure 1:**
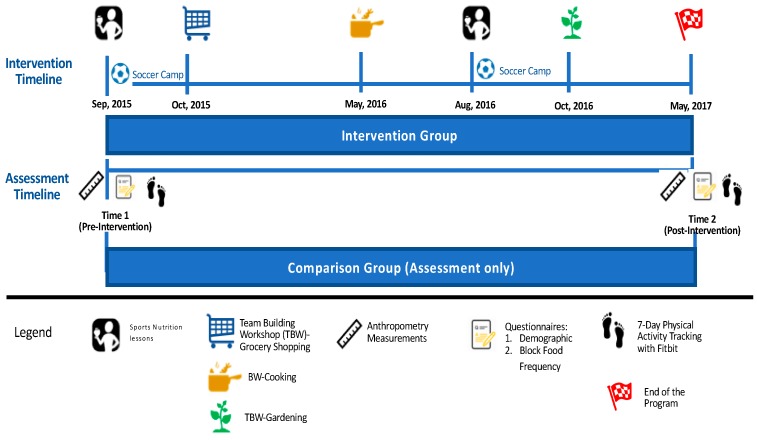
The WAVE program intervention experimental design (2015–2017) specific for diet and physical activity data.

The WAVE~Ripples for Change: Obesity Prevention in Active Youth (WAVE) program is a two-year integrated (research, education and extension) obesity prevention intervention targeting HS soccer players (aged 14–19 years). The intervention was age-specific and included health assessments, nutrition and diet questionnaires, face-to-face sports nutrition lessons, team-building workshops (TBWs) and experiential learning. The WAVE educational objectives were to encourage PA outside of sport, and teach sport nutrition and life-skills (e.g., meal planning, shopping on a budget, food preparation/cooking skills, and gardening) to support sustainable healthy eating and life-long PA among HS soccer players. Eligibility criteria included: (1) age 14–19 years; (2) enrolled in HS soccer; (3) living with a parent/caregiver; (4) no medical conditions preventing a normal diet; (5) internet access during the two-year study; and (6) proficiency in English. Figure 1 shows the experimental design of WAVE program for this study, see the full WAVE experimental design in Supplement Figure S1. See Manore et al [6] for more details on baseline sports nutrition knowledge and Meng et al [14] for details on program process evaluation. This manuscript only presents the two-year intervention data for changes in diet (FV, added sugar, saturated fat) and PA (steps/day; minutes of moderate-to-vigorous PA (MVPA)). All study instruments, protocols, and consent procedures were approved by the Oregon State University Institutional Review Board (#6317).

### 2.2. Recruitment and Participants

We used a two-step recruitment process. First, soccer coaches and their schools were recruited through OSU 4-H Soccer, then soccer players were recruited through their coaches and parents recruited at soccer parent meetings. Soccer teams were then assigned (non-randomized) to either the intervention (*n* = 278; 9 schools,) or comparison (*n* = 110; 3 schools) groups based on geographical location.

The WAVE program recruited 864 HS soccer players and 72% were enrolled after submitting youth assent and parent consent forms [14]. For data analyses using only baseline (time 1) data, we included only those soccer players (*n* = 388; 14–18 years; 13 schools; 24 soccer teams) who completed a demographic/health history/soccer experience questionnaire, diet and anthropometry assessments, and PA using Fitbit-zip for at least two days. For data analyses examining changes over the two-year intervention, we included 202 participants who completed both assessments periods (time 1, time 2) and 97 participants who completed PA assessments at time 1 and time 2.

### 2.3. Intervention Delivery

The majority of the intervention (lessons, TBW, PA measurement) was delivered to teams during the fall soccer season and summer soccer camps. The WAVE HS sport nutrition curriculum was delivered to teams and their coaches by a registered dietitian nutritionist (RDN) trained in sport nutrition and experienced in collegiate/professional soccer. Prior to intervention, all lessons were pilot-tested and revised based on athletes and input from experts. During year 1 the sport nutrition topics covered were hydration and pre/during/post-exercise fueling, while year 2 focused on body composition/image; maintaining muscle and staying healthy, and eating-well while eating out. PA outside of soccer practice and out-of-soccer season were encouraged. Newsletters reinforced lessons and provided recipes or tips to meet sport nutrition needs. Life-skills trainings were taught via three TBWs focused on menu planning, grocery shopping, cooking, knife skills and safety, and gardening, and delivered to teams by Extension faculty and community partners. These educational approaches all included experiential learning opportunities (e.g., food demonstrations and tastings, cooking with recipes high in FV, shopping on a budget, planning a post-game team meal, garden harvesting). The sports nutrition lessons and TBW encouraged intakes of FV, whole grains, and low-fat proteins, while selecting less processed foods and added sugar. See Supplement Table S1 for more information of the WAVE sports nutrition lesson curriculum and newsletter topics. 

### 2.4. Data Collection

Eligible participants enrolled in person or online. Questionnaires and assessments were completed at baseline (time 1) and post-intervention (time 2) (Figure 1). Based on the completion rate of intervention activities, each participant received gift cards (maximum = $65/year).

#### 2.4.1. Demographics, Parental Practices, and Anthropometry

The baseline questionnaire collected demographic (sex, age, races/ethnicity), NSLP participation, soccer experience, and youth perceived parental practices. Youth indicated how many days/week (0, 1–3, 4–5, or 6–7 days) their parent participated in the following behaviors: (1) making healthy food available at home; (2) role modeling healthy eating; (3) setting rules/expectations on what youth eat; (4) setting rules/expectation around screen time; and (5) role modeling PA. Anthropometry (height, weight; Tanita scale TM-300A, Tanita Corp., Singapore) assessments were completed at time 1 and time 2. Researcher completed required anthropometry training sessions to assure protocol fidelity and test/retest reliability.

#### 2.4.2. Dietary Assessment

A validated food intake screener (Block Fat/Sugar/Fruit/Vegetable Screener (Block-FSFV)) was used to measure youth’s FV, saturated fat, and added sugar intakes [15]. The screener reliability ranging from 0.70 to 0.78 over a four-month test/retest period [15]. Participants reported the portion size and consumption frequency of consuming 35 foods over the past week. Summary data included estimated average daily intakes for FV (cup equivalents), saturated fat (g/day) and added sugar (g/day). Estimated daily energy intake was only used to identify over/under-reporting as defined by Boucher et al [16]. 

#### 2.4.3. Physical Activity Assessment

At baseline (Time 1) and post-intervention (Time 2), all participants wore a Fitbit-Zip for 7-days. Fitbit-Zip correlates highly with the step data and MVPA data yielded by the ActiGraph among free-living adolescents [17]. This approach was pre-tested in the pilot study to assure viability and repeatability. Participants were given both oral and written instructions on how to use the Fitbit-zip. Daily texts/emails were sent to remind participants of wearing Fitbit. The comparison group’s Fitbit-Zip screen was covered with duct tape to avoid the self-monitoring effect on PA. Fitbit-Zip minute-by-minute data were aggregated into average daily steps, while MVPA was identified as time spent at >100 steps/min/day. Substantial empirical evidence supports using the >100 steps/min threshold for defining ambulatory time spent at or above a moderate-intensity in adults [18]. Recent research, using regression-based calibration in older adolescents (15–17 years), shows that this threshold (>100 steps/min) appears appropriate for defining time spent at or above a moderate-intensity in this age group [19]. All participants used in the PA analyses had at least two valid days of Fitbit-Zip measured PA (8-h/day wearing time) and daily values for steps/day and MVPA (min/day) were averaged across valid days. The Fitbit-Zip has shown excellent reliability (ICC = 0.90) for measuring steps in the laboratory setting [20] and previous research among a large and diverse sample of children and adolescents has demonstrated that two days of pedometer monitoring provides an acceptable level of reliability (ICC > 0.85) when estimating weekly steps/day [21]. Although there remains no consensus wear time threshold for defining a “valid day” when working with minute-by-minute Fitbit-Zip step data, we chose to employ the 8-h/day criteria utilized by a previous validation study in adolescents [17].

### 2.5. Data Analysis

At baseline (Time 1) descriptive statistics were calculated. Analysis of variance (ANOVA) models were used to examine mean baseline differences in FV, saturated fat, added sugar intake, average daily steps, and MVPA (min/day) between race/ethnicity (Latino/non-Latino, white and others), sex (female/male), and NSLP (participants/non-participants); interactions were also examined. Pearson correlations were used to evaluate associations between diet and PA variables and youth self-reported parental practices.

A series of analysis of covariance (ANCOVA) models, with categorical independent variables for group assignment, race/ethnicity, sex, and NSLP, were used to evaluate mean changes (time 2-time 1) in PA and diet intakes (FV, saturated fat, added sugar) between groups (intervention/comparison). For each outcome variable, a separate ANCOVA model was fitted with change score (time 2-time 1) as the dependent variable and baseline value as a covariate. Analyses were conducted using SPSS (version 24, SPSS, IBM SPSS, Inc., Chicago, IL, USA) and R Core Team.

## 3. Results

Table 1 provides the demographic characteristics of participants who completed baseline assessments. Participants were 15.3 ± 1.1 years of age and were normal weight based on average body mass index (BMI) percentile [22]. More specifically, 1% of participants were below 5th percentile, 77% were between 5th–85th percentile, 14.9% were between 86th–95th percentile, and 7% were greater than the 95th percentile. Overall, 38.1% of the youth athletes participated in the NSLP, with most (78.4%) being Latino; 45% engage in ≥ 2 sports, and 42% reported ≥ 1 injuries in the past year that needed ≥ 1-week of rest.

### 3.1. DietAssessment

For all participants at baseline (time 1), the average FV consumption was 2.8 cups/day (Table 1). There were no differences between sexes for FV and added sugar intakes, but females had significantly lower saturated fat intake than males (−4.4 g/day) (Table 2). Latinos had significantly higher added sugar intake than non-Latinos (+14.1 g/day), after adjusting for sex and NSLP. The two-year intervention resulted in participants significantly decreasing added sugar (−12g/day) and saturated fat (−2.7 g/day) intakes, with no changes in FV intake (Table 3). For the same time period, only saturated fat (−2.5 g/day) decreased in the comparison group. In comparing differences between groups over the two-year intervention, only added sugar intake was significantly lower in the intervention group (−10.4 g/day). This lower intake of sugar was attributed to a decrease in frequency (−11%) of consuming cake/cookie snack, ice cream, and ice-cream bars. There were no changes in sugar-sweetened beverage intake.

### 3.2. Physical Activity

For all participants (*n* = 388), baseline average steps/day were 9937 (males = 107,34 steps/day; females = 9353 steps/day) and MVPA was 33.3 min/day (Table 1). Neither the 2008 or 2018 Physical Activity Guidelines for Americans gives recommendation step guidelines for youth [23,24]. However, Adam et al. [25] translated the 2008 Physical Activity Guideline recommendation of 60 min/day of MVPA into step counts for youth 12–17 year (male ≥ 105,00 steps/day; female ≥ 9000 steps/day). Our participants met this step threshold recommendation. Overall, females were significantly less active (−1380 steps/day; −7.7 min MVPA/day) than males (Table 2). There were no differences in PA due to ethnicity or NSLP participation. During the two-year intervention, both groups had significantly higher PA in-season (time 1) compared to out-of-season (time 2; ~1500−2000 steps/day less) (Table 3). A similar response was observed in MVPA, with a higher level of MVPA (~17%) in-season vs. out-of-season. Over the two-year intervention, there were no significant differences between groups for PA.

**Figure 2:**
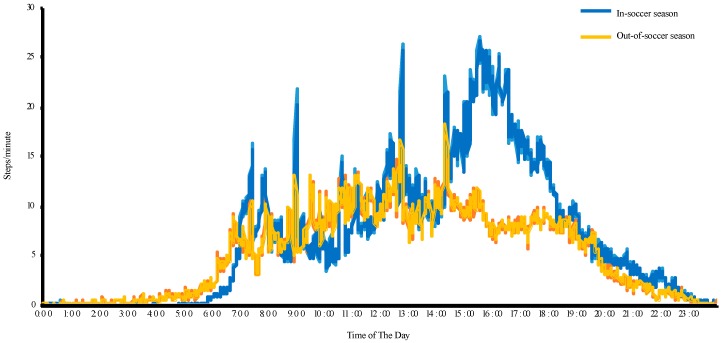
Daily pattern of steps-per-minute for all participants at baseline (in-soccer-season) and post-intervention (out-of-soccer season).

To further explore how participating in soccer contributed to meeting the PA guidelines, we calculated the average minute-by-minute steps for participants (*n* = 97; 2-day Fitbit data) using a 24-h scale (see Figure 2). Between 2:30–6:00 p.m., participants averaged 18.6 and 8.8 steps/minute in-season and out-season, respectively. During this time, athletes accumulated an extra of 2060 steps/day in-soccer season, which explains the in-/out-soccer season difference of 1820 steps/day (pre/post-intervention differences). 

### 3.3. Perceived Parental Support.

We also examined perceived parental support influences on youth’s diet and PA at baseline for all the participants combined (Table 4). Youth had higher FV intake when parents modeled healthy eating habits. Youth had lower added sugar intake when parent modeled healthy eating habits, made healthy foods available at home, and led physically active lifestyles.

## 4. Discussion

This is the first study to report changes in FV, saturated fat, and added sugar intakes, and PA in HS soccer players over a two-year intervention focused on teaching healthy eating behaviors, sport nutrition, lifetime PA, and life-skills for sport performance, health and obesity prevention. It is also the first study to evaluate whether diet and PA differ in HS athletes based on sex, race/ethnicity, social economic status, or youth self-reported parental practices.

### 4.1. Diet Assessment

Dietary assessments of FV, saturated fat, and added sugar intakes in HS soccer players are limited [26,27,28], and no published study has examined these factors in US Latino youth. The FV intakes in our participants (males = 2.8 cups/day; females = 2.7 cups/day) was higher than the mean FV intake reported in the 2013–2014 National Health and Nutrition Survey (NHANES) for adolescents age 12–18 years (1.9 cups/day) [29], but below the current Dietary Guideline for Americans (4.5–5 cups/day) (14–18 years) [30]. Three studies have examined differences in FV intake between youth sport participants and non-sport participants [31,32,33]. All found that sport participants had higher FV intake than non-sport participants, but no specific cup equivalents were reported. Others have examined the FV intake of youth athletes, including swimmers (1.6–2 cups/day) and rugby players (1.3 cups/day) [34,35], but no comparison group was included. Finally, Parnell et al [36] compared the FV intake of Canadian youth athletes to the Canadian diet recommendations for teens and found 40–54% met the FV guidelines of 7–8 servings per day. In contrast, only 13.9% of our participants met the Dietary Guidelines for Americans for FV intake [30]. 

For our participants, the average added sugar intake (males = 53 g/day; females = 44 g/day) was lower than that reported for 2013–2014 NHANES adolescents (81 g/day; 12–19 years) [29]. These differences may be due to methods used to assess added sugar intake. We used the Block-FSFV, which only includes 35 foods, while NHANES uses two 24-h recalls. Added sugar intake in our Latino soccer players was significantly higher (+14.1 g/day) than non-Latino soccer players. NHANES data (2003–2004) indicates that the major sources of added sugars of Latino youth were soda (28 g/day), fruit drinks (17 g/day), grain desserts (10 g/day), and candy (5 g/day) [37]. The high added sugar consumption in Latino youth has been attributed to acculturation and socioeconomic factors [38]. We, however, did not measure acculturation in our participants, so we cannot determine if acculturation affects added sugar intake. However, Manore et al. [6] found that Latino soccer players had lower sports nutrition knowledge (38.8%) compared to non-Latino players (48.4%), which may indicate lower general nutrition knowledge about added sugar intake.

Youth who self-reported that their parents frequently role modeled healthy eating habits and physically active lifestyles, and made healthy foods available at home had higher FV consumption, and lower added sugar intakes. These results support others who report that parental practices are important in shaping children’s nutrition behavior [39,40]. Ranjit et al. [39] found that increasing access and availability to healthy foods could increase middle school aged adolescents’ FV intake and decrease sugar-sweetened beverage intake. Battram et al. [40] also reported that parents were the primary gatekeepers and role models of children’s sugar beverage consumption. To our knowledge our research is the first to examine the influence of perceived parental support on FV and added sugar intake among HS athletes.

### 4.2. Physical Activity

Cross-sectional research shows that youth who participate in sports have higher MVPA (60 min/day) than non-sport participants (43 min/day) [41]. Yet, no study has explored the importance of helping active youth maintain their PA ‘out-of-sport-season’ or during other breaks from their sport participation. We followed HS soccer players for two years and analyzed their steps/day and MVPA both in/out-of-soccer season. Our results showed that the PA level in-soccer season met the 2008 Physical Activity Guidelines for Americans [23], but dropped below recommendations by 1820 steps/day during out-of-soccer season. During soccer season, participants generally have two games (1.5-h/each) and three/four practices (2-h/each) per week. Wearing a Fitbit is not allowed during games, but players typically accumulate 3–7 miles/game based on their position [42]. If these additional steps were added to the in-season steps, PA would be even higher than reported here and the differences in in-/out-soccer season PA would be even greater. These results confirmed the importance of school sports in promoting PA and the need to maintain youth’s PA when they are not engaged in organized sports. Although the WAVE project focused on active youth for obesity prevention, it is important to acknowledge that all youth need diet and PA life-skills. Thus, HS students who are not engaged in sports may need a different approach than used in this project to engage them in daily PA and learning life-skills around diet for obesity prevention. 

We found no association between youth PA (steps and MVPA) and youth self-reported parental practices. Research examining the influence of parental modeling of PA on children’s PA is equivocal, with some research being supportive [43,44], while others are not [45,46,47]. These data were all reported in young children (<12 years). No study has examined this association in active adolescents (≥14 years).

### 4.3. Strengths and Limitations

Our study had a number of strengths. First, we had a large sample (*n* = 388 at baseline; *n* = 202 completing pre/post assessments) of HS soccer players, with an average of 7.6-year playing experience; thus, they may be representative of many HS athletes who play at least one HS sport. Second, we had a diverse sample (58% female; 40% Latino; 40% NSLP participation), which allowed us to examine the association between diet and PA and sex, race/ethnicity, and social economic status. Third, the intervention lasted two years, compared to the 3–12 weeks interventions that are typically reported in the literature [48,49,50]. Finally, the WAVE intervention combining traditional face-to-face lessons and TBWs that included experiential learning that reinforced the lessons and allowed for practical application.

There were also limitations to this study. First, the Block-FSFV does not include Latino culturally-appropriate food or sport foods. The Block-FSFV was designed to focus on FV, saturated fat, and added sugar intake; thus, we could not calculate the percentage of energy from saturated fat and added sugar to compare to the 2015–2020 Dietary Guidelines for Americans recommendations. Second, dietary intake could have changed due to the time period (two years) we measured diet (pre-intervention: fall season 2015; post-intervention: spring season 2017). Typically, fall season is abundant in fruits and vegetables from the gardens and orchards, while spring is a time for celebration around graduation. However, there were no significant changes in FV intake across group or over time. Third, we did not measure the acculturation level, but all participants spoke English. Finally, the comparison group participants could have removed the duct-tape and monitored their daily steps, but we did not see differences between groups for steps in-/out-of-soccer season.

## 5. Conclusions

This is the first study to engage HS soccer players in a two-year obesity prevention program targeting sport nutrition education and healthy eating behaviors for growth, performance and health, and emphasizing the importance of daily PA and learning life-skills. Overall, the FV intakes were maintained over the intervention but were below recommendations, yet higher than those typically reported in the literature for youth and youth athletes. The intervention significantly decreased added sugar intake, by lowering the frequency of selecting cake/cookies and ice cream foods. Our lessons emphasized eating whole grains, FV, and lean protein sources and selecting food with less saturated fat and added sugar. Incorporating these eating behaviors into their lifestyle could lower added sugar intake. We found parents who modeled a healthy lifestyle, and made healthy foods available at home, had adolescents who reported lower added sugar intakes. Thus, parent involvement is important in shaping the healthy lifestyles of adolescents. Finally, we found PA recommendations were met during soccer season, but not out-of-season. Thus, it is important to engage active youth throughout the school year, helping them make daily PA a priority in their lifestyle. Based on our experience, self-monitoring alone is not enough to promote PA among active youth when they are not engaged in sport. Future studies should focus on maintaining PA in youth athletes when they are not engaged in sport, thus, helping them make the transition to being physically active adults.

## Figures and Tables

**Table 1:** Baseline characteristics of participants who completed demographic questionnaire, diet and anthropometry assessments, and physical activity using Fitbit-zip (*n* = 388).

Baseline Characteristics	All (*n* = 388)	Intervention Group (*n* = 278)			Comparison Group (*n* = 110)	
		Female (*n* = 152)	Male (*n* = 126)		Female (*n* = 72)	Male (*n* = 3 8)
Age (year), mean (SD) ^a^	15.3 (1.1)	15.1 (1.0)		15.3 (1.2)	15.4 (1.3)	15.9 (1.0)
Race, *n* (%)						
Latino	149 (38.4%)	43 (28.3%)		59 (46.8%)	34 (47.2%)	13 (34.2%)
Non-Latino ^b^	239 (61.6%)	109 (71.7%)		67 (53.2%)	38 (52.8%)	25 (65.8%)
NSLP ^c^, *n* (%)	148 (38.1%)	43 (28.3%)		53 (42.1%)	35 (48.6%)	17 (44.7%)
Prepare meal for themselves, *n* (%)	229 (59.0%)	99 (65.1%)		66 (52.4%)	44 (61.1%)	20 (52.6%)
Mean (SD) ^a^						
BMI ^d^ percentile	62.8 (25.0)	62.3 (23.5)		56.1 (27.5)	73.7 (21.0)	62.7 (21.0)
Years play soccer (year)	7.6 (3.7)	8.2 (3.3)		8.0 (3.7)	5.8 (3.6)	6.6 (4.0)
Fruit and vegetable (FV) intakes (cup equivalent/day)	2.8 (1.6)	2.9 (1.6)		2.8 (1.6)	2.4 (1.5)	2.7 (1.6)
Saturated fat (g/day)	21.8 (10.4)	19.8 (9.5)		24.1 (11.6)	20.9 (9.2)	23.6 (10.0)
Added sugar (g/day)	47.9 (38.3)	38.2 (30.6)		53.4 (43.7)	56.5 (38.4)	51.6 (39.9)
Daily steps/day	9937 (3180)	9019 (2534)		10629 (3551)	10061(2997)	11080(3642)
Moderate-to-vigorous physical activity (MVPA) (min/day)	33.3 (15.5)	29.2 (12.2)		38.3 (18.0)	31.8 (14.0)	35.9 (15.7)

^a^ Standard Deviation (SD); ^b^ Non-Latino includes participants who self-identified as White, American Indian/Alaska Native, Asian/Pacific Islander, or Black/African American; ^c^ NSLP = National School Lunch Program, participation indicates social economic status; ^d^ BMI = Body mass index (kg/m^2^), 5th percentile to less than the 85th percentile is considered normal or healthy weight [22].

**Table 2:** Correlations between dietary and physical activity variable and sex, ethnicity and participation in NSLP at baseline (*n* = 388).

Variable	Fruits and Vegetables (cups/day)	Saturated Fat (g/day)	Added Sugar (g/day)	Averaged Daily Step Counts (steps/day)	Moderate-to-Vigorous Physical Activity (min/day)
Mean difference (95% confident interval)					
Female ^a^	−0.11 (−0.42, 0.21)	−4.41 (−6.17, −2.06) **	−6.91 (−14.45, 0.58)	−1380(−2013, −747) **	−7.67 (−10.73, −4.62) **
Latino ^b^	−0.37 (−0.79, 0.05)	−1.73 (−4.46, 1.0)	14.08 (4.12, 24.03) **	−132 (−972, 706)	−0.07 (−4,11, 3.98)
NSLP ^c^	−0.30 (−0.71, 0.12)	−1.48 (−4.21, 1.24)	7.52 (−2.43, 17.48)	156 (−682, 995)	1.36 (−2.68, 5.40)

* Group are significantly different (** *p* < 0.01). ^a^ Females are compared with males; ^b^ Latinos are compared with non-Latino (White, American Indian/Alaska Native, Asian/Pacific Islander, or Black/African American); ^c^ National School Breakfast/Lunch Program (NSLP) participants are compared with non-NSLP percipients.

**Table 3:** Pre/post-intervention changes in dietary intake and physical activity (PA) within and between groups over the WAVE two-year intervention (*n* = 202).

Variable	Sample Size (*n*)	Baseline (in-Season) Mean (SD ^a^)	Post-Intervention (Out-of-Season) Mean (SD)	Within Group Changes (Post-Pre) ^b^	Intervention vs. Comparison Changes ^b^	*p*-Value ^c^
Fruit and vegetables (cup equivalent/day)	Intervention (*n* = 143)	2.7 (1.5)	2.6 (1.4)	−0.1 (−0.3, 0.1)	0.2 (−0.2, 0.5)	0.44
	Comparison (*n* = 59)	2.5 (1.5)	2.3 (1.2)	−0.2 (−0.6. 0.1)		
Saturated fat (g/day)	Intervention (*n* = 143)	21.2 (10.0)	18.7 (9.0)	−2.7 (−4.0, −1.4) *	−0.2 (−2.5, 2.2)	0.89
	Comparison (*n* = 59)	22.3 (9.4)	19.4 (9.0)	−2.5 (−4.5, −0.5) *		
Added sugar (g/day)	Intervention (*n* = 143)	43.6 (34.4)	33.7 (28.2)	−12.1 (−16.8, −7.4) *	−10.4 (−19.1, −1.6) *	0.02
	Comparison (*n* = 59)	57.6 (37.5)	50.6 (41.5)	−1.7 (−9.0, 5.5)		
Physical activity (average steps/day)	Intervention (*n* = 64)	9970 (3145)	7660 (3022)	−2058 (−2770, −1345) *	−473 (−1721, 775)	0.45
	Comparison (*n* = 33)	9849 (2977)	8752 (3186)	−1585 (−2587, −582) *		
Moderate-to-vigorous physical activity (MVPA) (min/day)	Intervention (*n* = 64)	34.3 (17.0)	24.3 (15.3)	−8.3 (−12.2, −4.5) *	−1.6 (−8.3, 5.2)	0.65
	Comparison (*n* = 33)	30.9 (13.2)	27.2 (16.8)	−6.8 (−12.2, −1.3) *		

* *p* value < 0.05; ^a^ = Standard Deviation (SD); ^b^ adjusted for the baseline value and wear time changes; ^c^ *p*-value for intervention vs. comparison.

**Table 4:** Correlations between diet and physical activity and youth self-reported parental practice at baseline (*n* = 388).

Variable	Fruits and Vegetables (cups/day)	Saturated Fat (g/day)	Added Sugar (g/day)	Averaged Daily Step Counts (steps/day)	Moderate−to−Vigorous Physical Activity (MVPA) (min/day)
Pearson’s correlation coefficient					
Parents make healthy food available, day/week ^a^	0.30 ***	0.09	−0.14 **	−0.02	−0.06
Parents role model healthy eating, day/week	0.23 ***	0.03	−0.11 *	−0.02	−0.04
Parents set rules on what youth eat, day/week	0.15 **	0.05	−0.10	0.03	0.02
Parents set rules on screen time, day/week	0.20 ***	0.03	−0.02	0.09	0.03
Parents role model PA, day/week	0.18 ***	0.02	−0.11 *	−0.01	−0.01

* Group are significantly different (* *p* < 0.05; ** *p* < 0.01; *** *p* < 0.001). ^a^ Youth self-reported the number of days in a week their parents have above parental practices, positive values indicant positive associations between days of parental practices and FV/saturated fat/added sugar/averaged daily steps/moderate-to-vigorous PA; negative value means the opposite.

## Supplementary Materials

The following are available online at http://www.mdpi.com/2072-6643/10/7/947/s1, Figure S1: Expanded experimental design, Table S1: Sports nutrition lessons.

## References

[1] Hales C.M., Carroll M.D., Fryar C.D., Ogden C.L. (2017). Prevalence of Obesity among Adults and Youth: United States, 2015–2016. https://www.cdc.gov/nchs/products/databriefs/db288.htm.

[2] National Center for Education Statistics Back to School Statistics for 2017. https://nces.ed.gov/fastfacts/display.asp?id=372.

[3] The National Federation of State High School Associations Participation Statistics: 2016–2017 High School Athletics Participation Survey Results. http://www.nfhs.org/ParticipationStatistics/PDF/2016-17_Participation_Survey_Results.pdf.

[4] Colo Rapids Youth Soccer (2016). US Youth Soccer Player Statistics. http://rapidsyouthsoccer.org/us-youth-soccer-player-statistics/.

[5] Turner R.W., Perrin E.M., Coyne-Beasley T., Peterson C.J., Skinner A.C. (2015). Reported sports participation, race, sex, ethnicity, and obesity in US adolescents from NHANES physical pctivity (PAQ_D). Glob. Pediatr. Health.

[6] Manore M.M., Patton-Lopez M.M., Meng Y., Wong S.S. (2017). Sport nutrition knowledge, behaviors and beliefs of high school soccer players. Nutrients.

[7] Janssen I., Boyce W.F., Simpson K., Pickett W. (2006). Influence of individual- and area-level measures of socioeconomic status on obesity, unhealthy eating, and physical inactivity in Canadian adolescents. Am. J. Clin. Nutr..

[8] Ogden C.L., Lamb M.M., Carroll M.D., Flegal K.M. (2010). Obesity and socioeconomic status in children and adolescents: United States, 2005–2008. NCHS Data Brief.

[9] Stalsberg R., Pedersen A.V. (2010). Effects of socioeconomic status on the physical activity in adolescents: A systematic review of the evidence. Scand. J. Med. Sci. Sports.

[10] Isasi C.R., Rastogi D., Molina K. (2016). Health issues in Hispanic/Latino youth. J. Lat. Psychol..

[11] Khan L.K., Sobal J., Martorell R. (1997). Acculturation, socioeconomic status, and obesity in Mexican Americans, Cuban Americans, and Puerto Ricans. Int. J. Obes. Relat. Metab. Disord..

[12] Drewnowski A., Darmon N. (2005). Food choices and diet costs: an economic analysis. J. Nutr..

[13] Boutelle K.N., Fulkerson J.A., Neumark-Sztainer D., Story M., French S.A. (2007). Fast food for family meals: Relationships with parent and adolescent food intake, home food availability and weight status. Public Health Nutr..

[14] Meng Y., Wong S.S., Manore M.M., Patton-López M. (2018). WAVE~Ripples for change obesity two-year intervention in high school soccer players: process evaluation, best practices, and youth engagement. Nutrients.

[15] Lalonde I., Graham M., Slovinec D’Angelo M., Beaton L., Brown J., Block T. (2008). Validation of the Block Fat/Sugar/Fruit/Vegetable screener in a cardiac rehabilitation setting. J. Cardiopulm. Rehabil. Prev..

[16] Boucher B., Cotterchio M., Kreiger N., Nadalin V., Block T., Block G. (2006). Validity and reliability of the Block98 food-frequency questionnaire in a sample of Canadian women. Public Health Nutr..

[17] Schneider M., Chau L. (2016). Validation of the Fitbit Zip for monitoring physical activity among free-living adolescents. BMC Res. Notes.

[18] Tudor-Locke C., Rowe D.A. (2012). Using cadence to study free-living ambulatory behaviour. Sports Med. Auckl. N. Z..

[19] Tudor-Locke C., Schuna J.M., Han H., Aguiar E.J., Larrivee S., Hsia D.S., Ducharme S.W., Barreira T.V., Johnson W.D. (2018). Cadence (steps/min) and intensity during ambulation in 6–20 year olds: The CADENCE-kids study. Int. J. Behav. Nutr. Phys. Act..

[20] Kooiman T.J.M., Dontje M.L., Sprenger S.R., Krijnen W.P., van der Schans C.P., de Groot M. (2015). Reliability and validity of ten consumer activity trackers. BMC Sports Sci. Med. Rehabil..

[21] Craig C.L., Tudor-Locke C., Cragg S., Cameron C. (2010). Process and treatment of pedometer data collection for youth: the Canadian Physical Activity Levels among Youth study. Med. Sci. Sports Exerc..

[22] Centers for Disease Control and Prevention Healthy Weight. https://www.cdc.gov/healthyweight/assessing/bmi/childrens_bmi/about_childrens_bmi.html.

[23] U.S. Department of Health and Human Services: 2008 Physical Activity Guidelines for Americans. https://health.gov/paguidelines/guidelines/children.aspx.

[24] U.S. Department of Health and Human Services 2018 Physical Activity Guidelines Advisory Committee Submits Scientific Report. https://health.gov/news/blog-bayw/2018/03/2018-physical-activity-guidelines-advisory-committee-submits-scientific-report/.

[25] Adams M.A., Johnson W.D., Tudor-Locke C. (2013). Steps/day translation of the moderate-to-vigorous physical activity guideline for children and adolescents. Int. J. Behav. Nutr. Phys. Act..

[26] Elizondo R.H.T., Bermudo F.M., Mendez R.P., Amorós G.B., Padilla E.L., de la Rosa F.J.B. (2015). Nutritional intake and nutritional status in elite Mexican teenagers soccer players of different ages. Nutr. Hosp..

[27] Ruiz F., Irazusta A., Gil S., Irazusta J., Casis L., Gil J. (2005). Nutritional intake in soccer players of different ages. J. Sports Sci..

[28] dos Santos D., da Silveira J.Q., Cesar T.B. (2016). Nutritional intake and overall diet quality of female soccer players before the competition period. Rev. Nutr..

[29] Bowman S., Clemens J.C., Friday J.E., Lynch K.L., LaComb R.P., Moshfegh A.J. (2017). Food Patterns Equivalents Intakes by Americans: What We Eat in America, NHANES 2003–2004 and 2013–2014.

[30] U.S. Department of Health and Human Services 2015–2020 Dietary Guidelines for Americans, 8th ed.. http://www.health.gov/dietaryguidelines/2015/guidelines/.

[31] Cavadini C., Decarli B., Grin J., Narring F., Michaud P.-A. (2000). Food habits and sport activity during adolescence: Differences between athletic and non-athletic teenagers in Switzerland. Eur. J. Clin. Nutr..

[32] Harrison P.A., Narayan G. (2003). Differences in behavior, psychological factors, and environmental factors associated with participation in school sports and other activities in adolescence. J. Sch. Health.

[33] Pate R.R., Heath G.W., Dowda M., Trost S.G. (1996). Associations between physical activity and other health behaviors in a representative sample of US adolescents. Am. J. Public Health.

[34] Collins A.C., Ward K.D., Mirza B., Slawson D.L., McClanahan B.S., Vukadinovich C. (2012). Comparison of nutritional intake in US adolescent swimmers and non-athletes. Health.

[35] Smith D.R., Jones B., Sutton L., King R.F.G.J., Duckworth L.C. (2016). Dietary intakes of elite 14- to 19-year-old English academy rugby players during a pre-season training period. Int. J. Sport Nutr. Exerc. Metab..

[36] Parnell J.A., Wiens K.P., Erdman K.A. (2016). Dietary intakes and supplement use in pre-adolescent and adolescent Canadian athletes. Nutrients.

[37] Reedy J., Krebs-Smith S.M. (2010). Dietary sources of energy, solid fats, and added sugars among children and adolescents in the United States. J. Am. Diet. Assoc..

[38] Pérez-Escamilla R., Putnik P. (2007). The Role of acculturation in nutrition, lifestyle, and incidence of type 2 diabetes among Latinos. J. Nutr..

[39] Ranjit N., Evans A.E., Springer A.E., Hoelscher D.M., Kelder S.H. (2015). Racial and ethnic differences in the home food environment explain disparities in dietary practices of middle school children in Texas. J. Nutr. Educ. Behav..

[40] Battram D.S., Piché L., Beynon C., Kurtz J., He M. (2016). Sugar-sweetened beverages: Children’s perceptions, factors of influence, and suggestions for reducing intake. J. Nutr. Educ. Behav..

[41] Silva G., Andersen L.B., Aires L., Mota J., Oliveira J., Ribeiro J.C. (2013). Associations between sports participation, levels of moderate to vigorous physical activity and cardiorespiratory fitness in childrenand adolescents. J. Sports Sci..

[42] Buchheit M., Mendez-Villanueva A., Simpson B.M., Bourdon P.C. (2010). Match running performance and fitness in youth soccer. Int. J. Sports Med..

[43] Hutchens A., Lee R.E. (2018). Parenting Practices and Children’s Physical Activity: An Integrative Review. J. Sch. Nurs. Off. Publ. Natl. Assoc. Sch. Nurses.

[44] Lloyd A.B., Lubans D.R., Plotnikoff R.C., Morgan P.J. (2015). Paternal Lifestyle-Related Parenting Practices Mediate Changes in Children’s Dietary and Physical Activity Behaviors: Findings From the Healthy Dads, Healthy Kids Community Randomized Controlled Trial. J. Phys. Act. Health.

[45] Jago R., Davison K.K., Brockman R., Page A.S., Thompson J.L., Fox K.R. (2011). Parenting styles, parenting practices, and physical activity in 10- to 11-year olds. Prev. Med..

[46] Chiarlitti N., Kolen A. (2017). Parental influences and the relationship to their children’s physical activity levels. Int. J. Exerc. Sci..

[47] Erkelenz N., Kobel S., Kettner S., Drenowatz C., Steinacker J.M., Research Group “Join the Healthy Boat—Primary School” (2014). Parental activity as influence on children’s BMI percentiles and physical activity. J. Sports Sci. Med..

[48] Elliot D.L., Moe E.L., Goldberg L., DeFrancesco C.A., Durham M.B., Hix-Small H. (2006). Definition and outcome of a curriculum to prevent disordered eating and body-shaping drug use. J. Sch. Health.

[49] Laramée C., Drapeau V., Valois P., Goulet C., Jacob R., Provencher V., Lamarche B. (2017). Evaluation of a theory-based intervention aimed at reducing intention to use restrictive dietary behaviors among adolescent female athletes. J. Nutr. Educ. Behav..

[50] Nascimento M., Silva D., Ribeiro S., Nunes M., Almeida M., Mendes-Netto R. (2016). Effect of a nutritional intervention in athlete’s body composition, eating behaviour and nutritional knowledge: A comparison between adults and adolescents. Nutrients.

