# Supplementary material for: Promoting Healthy Diet, Physical Activity, and Life-Skills in High School Athletes: Results from the WAVE Ripples for Change Childhood Obesity Prevention Two-Year Intervention
Source: Nutrients. 2018 Jul 23;10(7):947. doi: 10.3390/nu10070947 (PMC6073385; doi:10.3390/nu10070947)
Supplement: Supplementary file 1 [file nutrients-10-00947-s001.zip › Supplementary/Figure S1 expanded experimental design.pptx]

## Slide 1
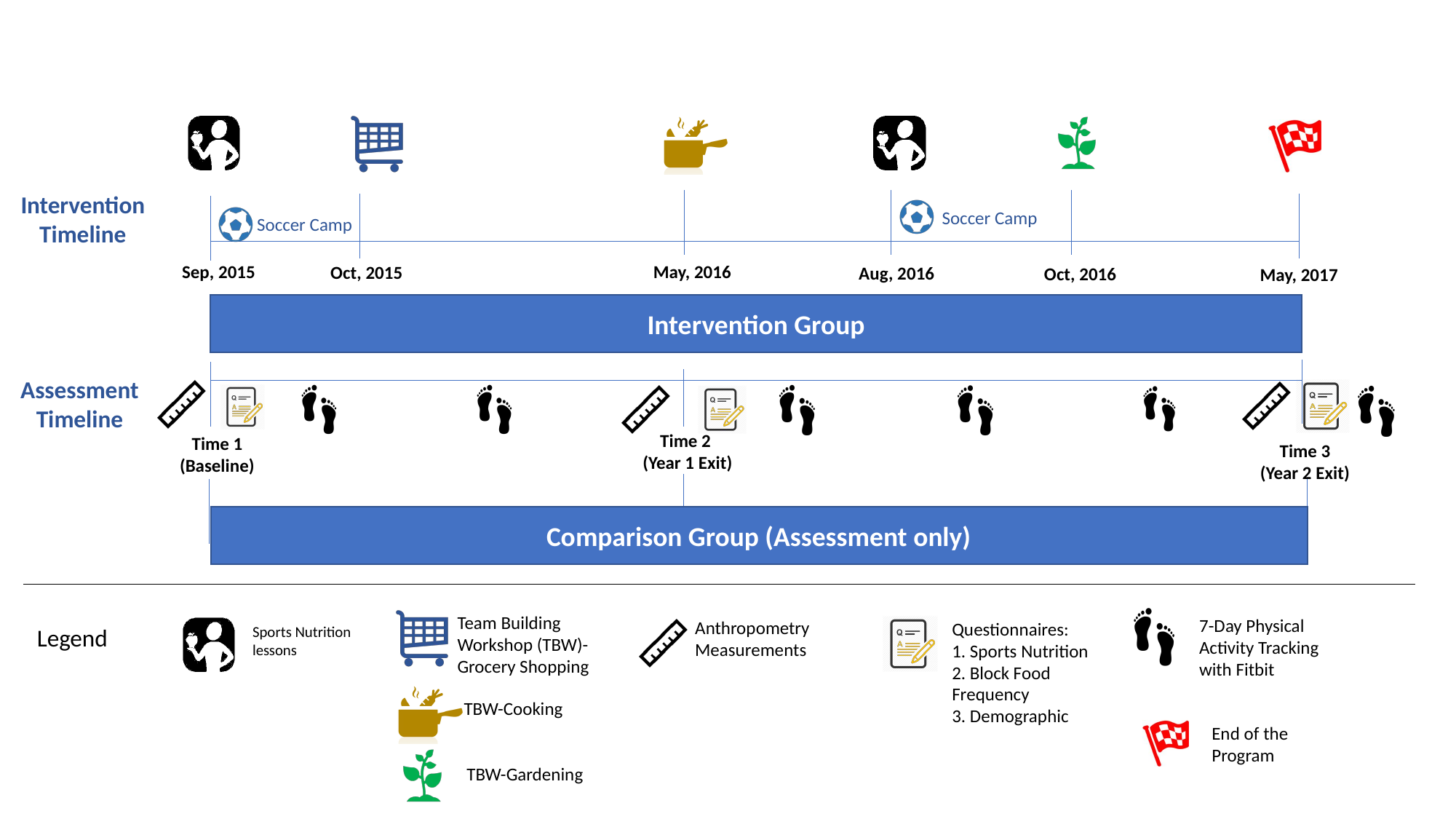

Intervention Timeline
Soccer Camp
Soccer Camp
Sep, 2015
May, 2016
Oct, 2015
Aug, 2016
Oct, 2016
May, 2017
Intervention Group
Assessment Timeline
Time 2
(Year 1 Exit)
Time 1 (Baseline)
Time 3
(Year 2 Exit)
Comparison Group (Assessment only)
Team Building Workshop (TBW)- Grocery Shopping
7-Day Physical Activity Tracking with Fitbit
Anthropometry Measurements
Questionnaires:
1. Sports Nutrition 2. Block Food Frequency
3. Demographic
Sports Nutrition lessons
Legend
TBW-Cooking
End of the Program
TBW-Gardening
