# Supplementary material for: Promoting Healthy Diet, Physical Activity, and Life-Skills in High School Athletes: Results from the WAVE Ripples for Change Childhood Obesity Prevention Two-Year Intervention
Source: Nutrients. 2018 Jul 23;10(7):947. doi: 10.3390/nu10070947 (PMC6073385; doi:10.3390/nu10070947)
Supplement: Supplementary file 1 [file nutrients-10-00947-s001.zip › Supplementary/Table S1.docx]

Table S1. Sport nutrition lessons.

| **Lesson** | **Title** | **Lesson Goals** |
| --- | --- | --- |
| **1** | **Hydration** | To provide athletes with the knowledge to understand the purpose of proper hydration for sport and exercise, and give them the tools and skills needed to implement a hydration plan to delay onset of dehydration, improve and maintain training and performance, and decrease risk of illness and injury related to dehydration.  Newsletter: Hydration. |
| **2** | **Pre-Exercise Fueling** | To provide athletes with the knowledge necessary to understand the purpose of proper fueling prior to exercise, and give them the tools and skills necessary to implement a pre-exercise fueling plan to delay onset of fatigue and dehydration, improve and maintain training and performance, and avoid GI (gastrointestinal) discomfort.  Newsletter: Pre-exercise |
| **3** | **During Exercise Fueling** | To provide athletes with the knowledge to understand the purpose of proper fueling during exercise, and give them the tools and skills necessary to implement a during-exercise fueling plan to improve and maintain training and performance, and avoid GI (gastrointestinal) discomfort. |
| **4** | **Recovery Nutrition** | To provide athletes with the knowledge to understand the purpose of proper recovery nutrition and the skills to implement a recovery nutrition plan to: replete glycogen stores, rehydrate, initiate recovery and adaptation processes in the body that require carbohydrate, protein, and water, and achieve the maximum gains from training to maintain and/or improve performance.  Newsletter: Protein for performance (males).  Newsletter: Protein for performance (females). |
| **5** | **Body Composition and Image** | Lessons were different for males and females.  Male: To provide athletes with an understanding of body composition, how it is measured, and factors that influence body size and composition.  Females: To provide athletes with an understanding of body composition and a heathy perspective on body image in order to develop body appreciation and acceptance. |
| **6** | **Maintaining Muscle and Staying Well** | To provide athletes with the knowledge to understand how nutrient timing and composition of foods/meals will help them meet their protein needs; maintain a strong immune system and stay healthy while participating in sports. |
| **7** | **Eating Well while Eating Out** | Help athletes understand how to make better food selections outside the home that are within their budgets. Raise awareness that cooking and eating food from home can be the most affordable and 'healthy' option. |
